# Structurally Flexible 2D Spacer for Suppressing the Electron–Phonon Coupling Induced Non-Radiative Decay in Perovskite Solar Cells

**DOI:** 10.1007/s40820-024-01401-9

**Published:** 2024-04-24

**Authors:** Ruikun Cao, Kexuan Sun, Chang Liu, Yuhong Mao, Wei Guo, Ping Ouyang, Yuanyuan Meng, Ruijia Tian, Lisha Xie, Xujie Lü, Ziyi Ge

**Affiliations:** 1 Zhejiang Provincial Engineering Research Center of Energy Optoelectronic Materials and Devices, Ningbo Institute of Materials Technology and Engineering, Chinese Academy of Sciences, Ningbo, 315201 People’s Republic of China; 2 School of Materials Science and Chemical Engineering, Ningbo University, Ningbo, 315211 People’s Republic of China; 3 Center of Materials Science and Optoelectronics Engineering, University of Chinese Academy of Sciences, Beijing, 100049 People’s Republic of China; 4 Center for High Pressure Science and Technology Advanced Research (HPSTAR), Shanghai, 201203 People’s Republic of China

**Keywords:** Electron–phonon coupling, A-site cation engineering, Non-radiative recombination

## Abstract

This study presents experimental evidence of the dependence of non-radiative recombination processes on the electron–phonon coupling of perovskite in perovskite solar cells (PSCs). Via A-site cation engineering, a weaker electron–phonon coupling in perovskite has been achieved by introducing the structurally soft cyclohexane methylamine (CMA^+^) cation, which could serve as a damper to alleviate the mechanical stress caused by lattice oscillations, compared to the rigid phenethyl methylamine (PEA^+^) analog. It demonstrates a significantly lower non-radiative recombination rate, even though the two types of bulky cations have similar chemical passivation effects on perovskite, which might be explained by the suppressed carrier capture process and improved lattice geometry relaxation. The resulting PSCs achieve an exceptional power conversion efficiency (PCE) of 25.5% with a record-high open-circuit voltage (*V*_OC_) of 1.20 V for narrow bandgap perovskite (FAPbI_3_). The established correlations between electron–phonon coupling and non-radiative decay provide design and screening criteria for more effective passivators for highly efficient PSCs approaching the Shockley–Queisser limit.

**Supplementary Information:**

The online version contains supplementary material available at 10.1007/s40820-024-01401-9.

## Highlights

- The soft 2D material reduces the coupling strength between carriers and longitudinal optical phonons, releasing the mechanical stress of lattice vibration.
- The power conversion efficiency of rigid devices and flexible devices reaches 25.5% and 23.4%, respectively.

**Supplementary Information:**

The online version contains supplementary material available at 10.1007/s40820-024-01401-9.

## Graphical abstract

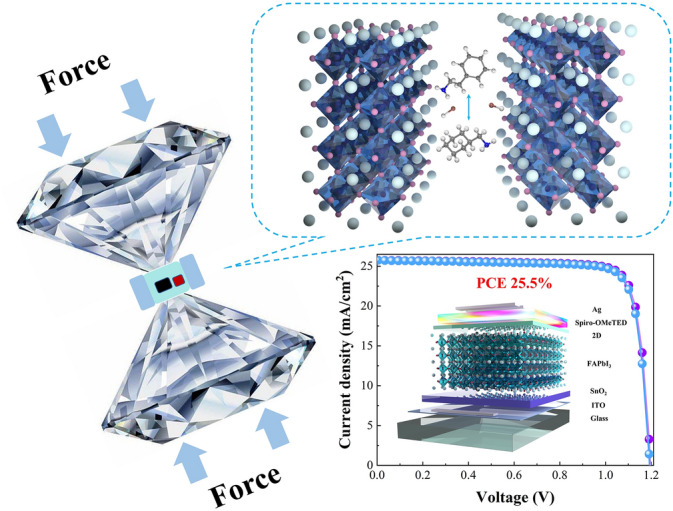

## Introduction

Perovskite solar cells have emerged as a promising candidate for future photovoltaic technology due to their remarkable power conversion efficiency and ease of fabrication at low temperatures. In the past decade, there have been continuous advancements in interfacial engineering [1–5], perovskite composition [6–8], and exploration of crystallization methods [9–11]. As a result, the power conversion efficiencies (PCEs) of perovskite solar cells (PSCs) have skyrocketed to 26% for rigid devices [12] and 24.5% for flexible PSCs [13], rivaling the PCEs achieved during the development of crystalline silicon solar cells over several decades. All the evidence above indicates that PSCs have promising potential for applications in centralized photovoltaic power stations for rigid PSCs and wearable/portable electronics for flexible PSCs (f-PSCs) [14].

The low-temperature solution process inevitably introduces defects into the perovskite films, forming Shockley–Read–Hall (SRH) electron–hole recombination centers. In organometallic halide perovskite thin films, the predominant defects are intrinsic point defects, such as interstitials (MA_i_, Pb_i_, and I_i_), vacancies (V_MA_, V_Pb_, and V_I_), and antisites (MA_Pb_, MA_I_, Pb_MA_, Pb_I_, I_MA_, and I_Pb_) [15, 16]. Most defects are typically found on the film surface or at grain boundaries. Passivations on both cationic and anionic vacancies have been extensively studied in the previous reports, which result in suppressed non-radiative recombination losses and improved device efficiency [17–19]. However, there is still a significant disparity between the efficiency of state-of-the-art devices and the theoretical limit set by the Shockley–Queisser theory. This could be because defect passivation primarily emphasizes chemical passivation with little consideration for the electron capture process by defect states during light illumination, which serves as the predominant pathway for voltage loss.

A few previous studies have suggested that the distortion of the lattice due to electron–phonon coupling affects the non-radiative recombination rate [20, 21]. In 2015, Zhu et al. observed the broad range of trap energies and the relative energetic positions of excitonic traps from photoluminescence (PL) spectra [22]. Thus, they proposed that the excitonic traps resulted from the self-trapping of band-edge excitons due to the electron–phonon coupling, rather than from common chemical defects, as the trap state energy would be well-defined. Sargent et al. also reported a faster non-radiative decay in the perovskite film with stronger vibrational overlap between the ground- and excited-state vibrational wave functions [23]. Recently, Walsh et al. utilized first-principle calculations to elucidate the underlying mechanism of electron–phonon coupling in the non-radiative recombination process. It was found that the charge capture coefficient increased due to the rotations of the inorganic octahedral cage upon illumination, which resulted in a strong coupling between the electronic charge state of the defect and the lattice distortion [24]. Several research groups independently concluded, through density functional theory (DFT) calculations, that low-frequency lattice phonons (from the Pb-I tensile vibration mode) are primarily responsible for the vibronic coupling between the valence band maximum (VBM) and conduction band minimum (CBM), which would increase the non-adiabatic coupling and result in non-radiative electron–hole recombination [21, 24–26]. Therefore, they proposed that suppressing of electron–phonon coupling would be an important factor in enhancing defect tolerance. However, to date, there is still a lack of experimental evidence supporting the correlations between electron–phonon coupling and non-radiative decay processes, as well as their effects on PSC efficiencies. This absence hinders further improvements in efficiency toward the S–Q limit.

One effective method for accommodating the electron–phonon coupling was proposed by Guo et al. by introducing a two-dimensional (2D) perovskite organic spacer with varying structural rigidities, achieved through the co-adaptation of the organic cations and inorganic frameworks [27]. In this study, organic cation engineering, which is an effective method for modulating the strength of electron–phonon coupling [28], is employed to mediate the electron capture process in perovskite film. Two representative bulky organic cations, cyclohexane methylamine and phenethyl methylamine, have been investigated in this study for their ability to form 2D phases in 2D/3D systems**.** The chair-like conformation and various vibration modes of the cyclohexane methylamine (CMA^+^) cation provide it a soft structure (as shown in Fig. S1); while in contrast, phenethyl methylamine (PEA^+^) exhibits significantly higher rigidity. By analyzing the Fröhlich coupling through cryogenic PL measurements, we have discovered a weaker interaction between carriers and phonons in the CMA-based 3D/2D perovskite compared to the PEA-based 3D/2D perovskite system. The weaker interaction may be attributed to the soft interlayer of CMA^+^ inducing damping effects on the inorganic perovskite lattices. In the evaluation of the non-radiative recombination process, it is quite intriguing to find that although the CMA- and PEA-based 3D/2D perovskite exhibit a similar density of trap states (measured in the dark), the non-radiative decay is faster in the latter system. The experiment verifies the correlation between electron–phonon coupling and the non-radiative decay in the perovskite system, providing new insights for the future design and screening of 2D structures. As a result, CMA-based 3D/2D PSCs have achieved an impressive PCE of 25.5% with excellent stability. Furthermore, the soft CMA^+^ interlayer makes the 2D perovskite promising for applications in f-PSCs, demonstrating a 23.4% PCE with exceptional mechanical flexibility.

## Experimental Sections

### Materials

SnO_2_ colloid precursor (tin (IV) oxide, 15% in H_2_O colloidal dispersion), *N,N*-dimethyl formamide (DMF, 99.8%), dimethyl sulfoxide (DMSO, 99.7%), and chlorobenzene (CB, 99.9%) were purchased from Beijing J&K Scientific Ltd. 2,2′,7,7′-tetrakis[*N,N*′-di(4-methoxyphenyl)amine]-9,9′-spirobifluorene (Spiro-OMeTAD, 99.8%), lithium bis (trifluoromethanesulfonyl) imide (Li-TFSI, 99%), cyclohexanemethylamine iodide (CMAI), phenethylamine methylamine iodide (PEAI), 4-tertbutylpyridine (TBP, 96%), and tris (2-(1H-pyrazol-1-yl)-4-tert-butylpyridine)-cobalt(III) tris [bis (trifluoromethyl sulfonyl) imide] (FK209, 99.5%) were purchased from Xi’an Polymer Light Technology Corporation. SnO_2_ colloid precursor (tin (IV) oxide, 15% in H_2_O colloidal dispersion), lead(II) iodide (PbI_2_, 99.99%) and formamidine iodide (FAI, 99.9%), and the ITO substrate were purchased from Advanced Election Technology Co., Ltd. All these commercially available materials were used directly as received without any further purification.

### Device Fabrication

The ITO substrate that had undergone partial etching was meticulously cleaned in a specific sequence, involving the use of detergent, deionized water, acetone, and isopropanol, each for a period of 30 min. After being thoroughly cleansed, the ITO/glass substrate was dried using N_2_ and then subjected to plasma treatment for 5 min. Subsequently, a SnO_2_ nanoparticle solution (with a 15% concentration in H_2_O colloidal dispersion) was prepared and diluted with deionized water to obtain a 7.5% concentration. The diluted SnO_2_ precursor solution was then spin-coated onto the ITO glass for 15 s at 6500 rpm and annealed at 150 °C for 30 min. The spin-coated electron transport layer was further treated with UV ozone for 10 min, followed by transferred to the glove box and spin-coated with 50 μL of FAPbI_3_ by two-step spin-coating procedure at speeds of 1000 rpm for 5 s and 5000 rpm for 20 s. The resulting film was annealed at 150 °C for 10 min in an environment with a humidity of 40%. As for the perovskite precursor solution, 1-mol PbI_2_ and 1-mol FAI were dissolved in 1500 μL of γ-valerolactone, after 24 h of stirring, the solution was filtered, then heated slowly to 150 °C in an oil bath. The resulting black FAPbI_3_ was subsequently washed with both acetonitrile and ether. One-mL perovskite precursor solution (DMF:DMSO = 4:1) was prepared by adding 35 mol% MACI into the synthesized 1-mol FAPbI_3_ black powder. For the deposition of 3D/2D films, CMAI/PEAI isopropyl alcohol solution was spin-coated onto the as-casted FAPbI_3_ film (without annealing) at 6000 rpm for 25 s, followed by annealing at 100 °C for 10 min. Spiro-OMeTAD film was then spin-coated on the film at 4000 rpm for 30 s. The Spiro-OMeTAD solution was composed of 72.5-mg Spiro-OMeTAD, 18 μL of LiTFSI stock solution (520 mg mL^−1^ in acetonitrile), 8 μL of FK209 solution (300 mg mL^−1^ in acetonitrile), and 28 μL of tBP. Finally, 100-nm Ag electrodes were thermally evaporated to complete the fabrication of the entire device. The effective area of all devices was 0.04 cm^2^.

### Device Characterization

The device's photovoltaic performance was evaluated using Newport Oriel Sol3A 450 W solar simulator's simulated solar lamp, which provided 100 mW cm^−2^ with AM 1.5 G. The *J–V* curves of the PSCs were analyzed at room temperature. The 450-W xenon lamp used as the light source was calibrated to 100 MW cm^−2^ using a silicon reference cell, thereby standardizing the procedure. Forward scanning (1.5 to − 0.4 V, step 0.02 V) and reverse scanning (from − 0.4 to 1.5 V, step 0.02 V) were the scanning parameters utilized, and a scan rate between 0.01 and 0.5 V s^−1^ was selected for an accurate measurement. Furthermore, the external quantum efficiencies (EQE) spectra were recorded using the solar cell quantum efficiency test system provided by Elli Technology, Taiwan. Regarding the analytical tools employed, Kratos Axial Super Dald was utilized to measure X-ray photoelectron spectroscopy (XPS), while SEM images were obtained via a scanning electron microscope (Verios G4 UC) from Rimono Scientific Company, USA, using 2 kV. The fluorescent spectra were recorded utilizing the Horiba Jobin Yvon Fluorolog-3 Spectrofluorometer system through photon excitation induced by incident light at 450 nm on the glass surface. Additionally, time-resolved fluorescence was analyzed using the Edinburgh instrument, while FLS 980 fluorescence spectrometer was deployed for TRPL decay excitation measurement. The quality of fitting for the function of two exponential models expressing attenuation is assessed with the parameters *χ*^2^ estimate (0.90 ≤ *χ*^2^ ≤ 1.10). The Chi 660e electrochemical measurement workstation from Chengdu Equipment Company, Shanghai, China, was employed to perform SCLC analysis under dark conditions. Finally, the C^−2^-V spectra were measured using the Chenhua CHI760E electrochemical workstation. Femtosecond transient absorption spectroscopy was measured by SOL-F-K-HP-T in 400 nm. The AFM measurements were carried out by Dimension ICON SPM (Dimension Icon, German).

Symmetrical diamond anvil cells, using Type II-a ultralow fluorescence diamonds with a culet size of 500 μm, were employed to create a high-pressure environment. To form the high-pressure sample chamber, a preindented T301 gasket with a thickness of approximately 50-μm and a 300-μm diameter hole in its center was laser-drilled. Single crystals were exfoliated into thin flakes and transferred onto the diamond culets. The ruby fluorescence method was used to determine the pressure, with mineral oil serving as the pressure transmitting medium. For the absorption measurements, a Xe lamp (EQ-99X-FC-S) was chosen as the white light source. For the PL measurement, a 405-nm continuous laser was used for excitation.

### Theoretical Calculation

The geometric optimization, electrical structure, and pressure calculations in this research are performed using the Vienna ab initio simulation software (VASP). To simulate electron exchange-related interactions, the Perdew–Burke–Ernzerhof (PBE) [29] functional is utilized, and the projection enhanced wave (PAW) [29] approach is used for electron–ion–nucleus interactions. The bandgap variations brought on by structural changes under pressure can still be utilized as a reference, even if PBE functionals may somewhat diminish the bandgap results due to the computational cost reductions. In order to handle van der Waals interactions in perovskites, we employ the Grimme DFT-D3 approach with Becke–Johnson damping [30, 31]. Geometry optimization is carried out with the Γ-centered 4 × 4 × 1 Monkhorst–Pack k-point mesh and the 400-eV plane wave energy cutoff. For density of states computations to produce an accurate electronic structure, a denser 8 × 8 × 2 k-point mesh is used. The geometric structure is regarded as convergent when the energy difference between all ions is smaller than -10^–5^ eV. After optimizing the geometric structure, a model with a step size of 0.2 GPa from 0 to 1 GPa was obtained by applying hydrostatic pressure to the system.

## Results and Discussion

### Characterization of Perovskite Films

**Fig. 1:**
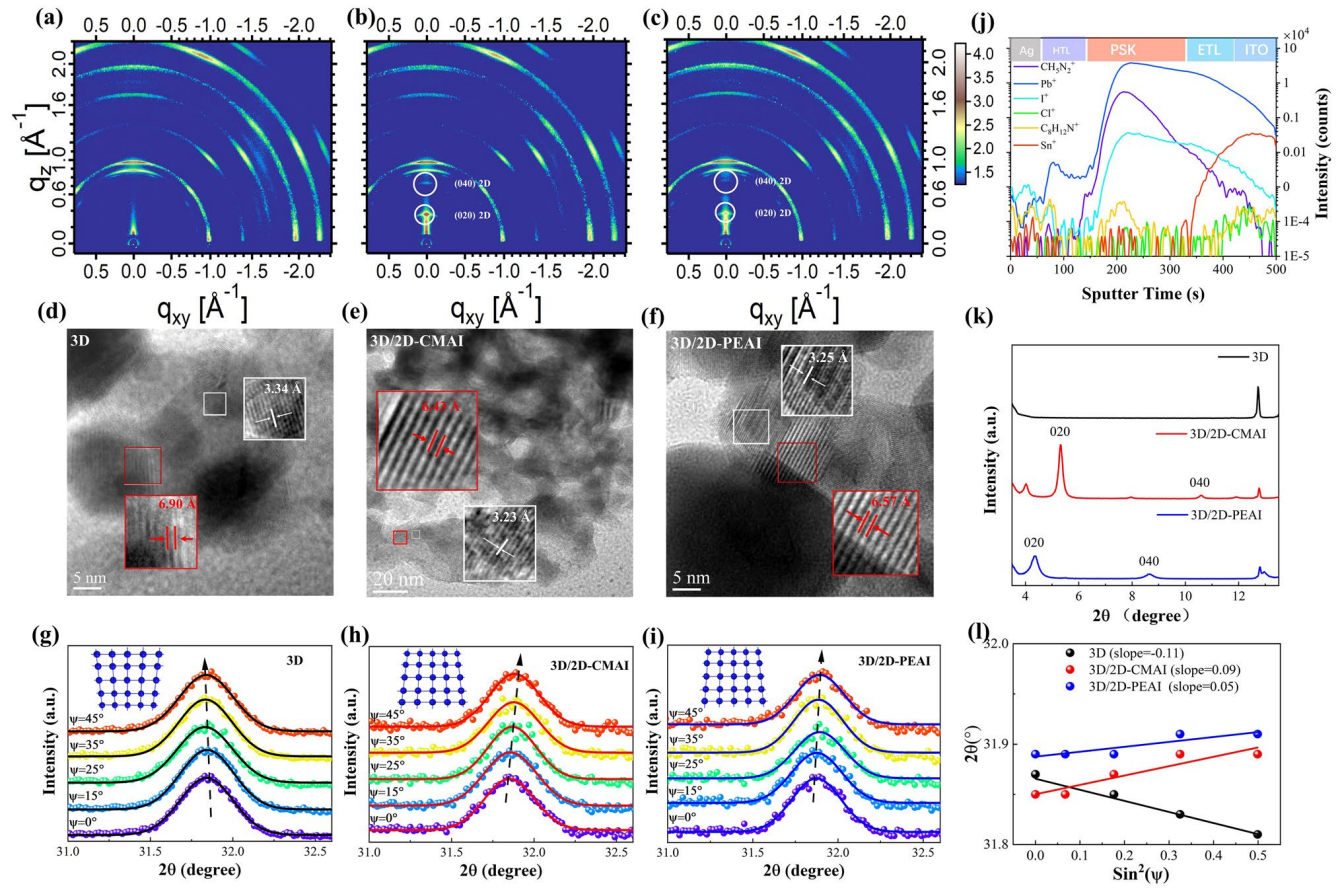
**a–c** GIWAXS patterns of 3D, 3D/2D-CMAI, and 3D/2D-PEAI films. **d–f** TEM images of 3D, 3D/2D-CMAI, and 3D/2D-PEAI films. **g–i** GIXRD patterns at different tilting angles of 3D, 3D/2D-CMAI, and 3D/2D-PEAI films. **j** ToF–SIMS depth profile for the 2D treated perovskite film deposited on ITO substrate. **k** X-ray diffraction patterns of 3–13° with 3D, 3D/2D-CMAI, and 3D/2D-PEAI films. **l** Linear fit of 2*θ*-sin^2^ (ψ) for 3D, 3D/2D-CMAI, and 3D/2D-PEAI films

The 3D and 3D/2D films were prepared following the fabrication process described in the experimental section of the Experimental Sections. The grazing incidence wide-angle X-ray scattering (GIWAXS) was first conducted to examine the crystallographic structures of 3D/2D-CMAI, 3D/2D-PEAI, and 3D perovskite films. According to the GIWAXS images shown in Fig. 1a–c, all three perovskite films exhibit obvious Debye–Scherrer diffraction rings at *q*_z_ = 1.0 Å^−1^, corresponding to the (100) 3D diffraction peak. For the 3D/2D films, diffractions appearing at scattering vector q_z_ values from 0.3 to 0.7 Å^−1^ are indexed to the (020) and (040) planes of the layered 2D phases (n = 2), respectively, as indicated in Fig. 1b ((CMA)_2_FAPb_2_I_7_) and Fig. 1c ((PEA)_2_FAPb_2_I_7_). In both 3D/2D films, the sharp 2D (020) diffraction peaks are indicative of highly oriented crystals in the vertical direction, as illustrated in Fig. S3a-c. Thus, fewer organic dielectric layers are spanned in the out-plane direction [32], which predicts a convenient carrier transport pathway. The X-ray diffraction (XRD) patterns displayed in Figs. 1k and S2 also verify the formation of 2D phases in the 3D/2D films, which are consistent with the GIWAXS results.

The high-resolution transmission electron microscopy was used to inspect the microstructural morphologies of the 3D and 3D/2D films, and the lattices were analyzed using the fast Fourier transform. We focus on the (100) and (200) crystal planes for analysis because of the better crystallization as shown in Fig. S2. From Fig. 1d-f, the spacing of the (100) crystal plane becomes narrower, decreasing from 6.90 Å for bare 3D to 6.43 Å for 3D/2D-CMAI and 6.57 Å for 3D/2D-PEAI. The crystal interplanar spacing at (200) experiences the same trend, decreasing from 3.34 Å for 3D to 3.23 Å for 3D/2D-CMAI and 3.25 Å for 3D/2D-PEAI. We speculate that the observed narrowing in the crystal spacing is attributed to the impact of 2D phases in alleviating the tensile stress between the 3D grains [33, 34]. To further discuss the regulation of film strain by 2D phases, the grazing incident X-ray diffraction (GIXRD) with 2θ-sin^2^ψ measurement was characterized. Figure 1g shows that as the angle ψ increases from 0° to 45°, the scattering peak of the pristine 3D shifts to a lower angle direction, indicating the presence of tensile stress in the 3D film. In contrast, Fig. 1h and i shows that the 3D/2D-CMAI and 3D/2D-PEAI exhibit the opposite trend, with the diffraction peaks shifting toward a higher degree as ψ increases. The linear fit of 2θ-sin^2^ψ of 3D, 3D/2D-CMAI, and 3D/2D-PEAI films in Fig. 1l shows slopes of -0.11, 0.09, and 0.05, respectively. It confirms that the 3D perovskite has relieved its tensile stress and transitioned into a slight compressive strain with the assistance of 2D phases, which is expected to benefit photovoltaic performance and device stability [35–37]. To gain a better understanding of the 2D phase distribution in 3D film, time-of-flight secondary ion mass spectrometry (ToF–SIMS) was conducted. The results in Fig. 1j demonstrate that the 2D phases are formed throughout the perovskite film, rather than being confined to the film surface. Therefore, we assume that the 2D phases would reside on the grain boundaries of the FAPbI_3_ perovskite across the entire film.

The surface film morphologies of 3D, 3D/2D-CMAI, and 3D/2D-PEAI films were subsequently investigated by scanning electron microscopy. It was noted that the 3D perovskite film is deposited from FAPbI_3_ single crystals by inverse temperature crystallization method [38, 39], resulting in uniform grains with a size of over 1 μm, as shown in Fig. S4. This is crucial for the fabrication of highly efficient PSCs. The film morphology is significantly altered due to the recrystallization of the 3D perovskites during the organic cation treatments, as the grains and grain boundaries of the 3D perovskites become fully covered with 2D phases. As shown by the atomic force morphology images in Fig. S5, both 2D treatments result in decreased root-mean-square roughness of the film surface, which not only acts as a more effective defect “passivator”, but also creates a protective layer to prevent moisture evasion from destabilizing the PSCs. The higher water contact angles of the 3D/2D films compared to those of the 3D film shown in Fig. S6 are indicative of the stronger hydrophobicity, thus higher ambient stability of the film.

### Theoretical Calculations

**Fig. 2:**
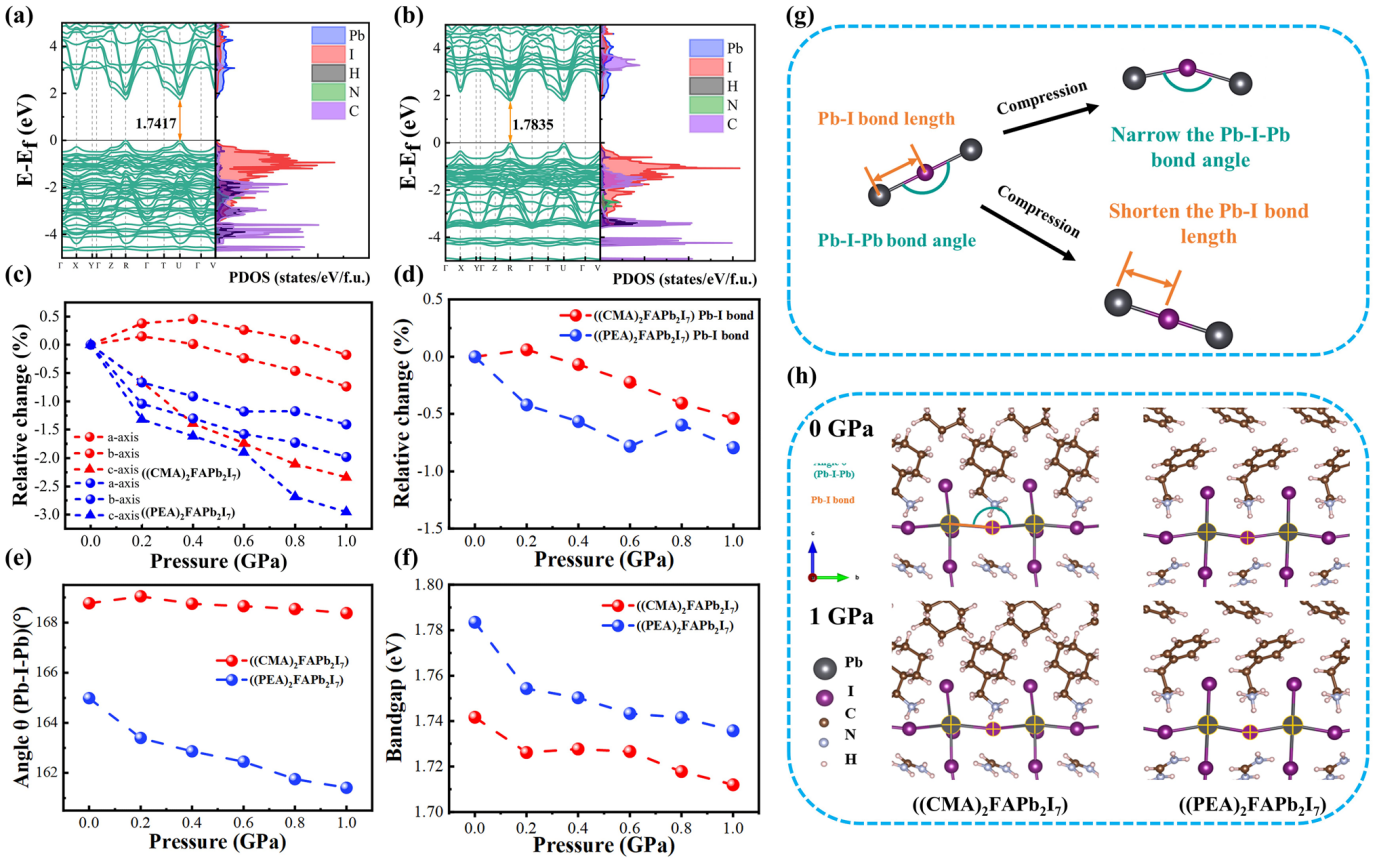
**a, b** Theoretical energy band structure and density of states of (CMA)_2_FAPb_2_I_7_ and (PEA)_2_FAPb_2_I_7_. **c** Relative changes of lattice parameters of (CMA)_2_FAPb_2_I_7_ and (PEA)_2_FAPb_2_I_7_ with pressure change. **d** Relative changes of the Pb-I bond length of (CMA)_2_FAPb_2_I_7_ and (PEA)_2_FAPb_2_I_7_ with pressure change. **e** Variation of the Pb-I-Pb bond angle of (CMA)_2_FAPb_2_I_7_ and (PEA)_2_FAPb_2_I_7_ with pressure change. **f** Variation of the bandgap of (CMA)_2_FAPb_2_I_7_ and (PEA)_2_FAPb_2_I_7_ with pressure change. **g** Schematic diagram of bond length change and bond angle change between metal and halide. **h** Structural optimization models of (CMA)_2_FAPb_2_I_7_ and (PEA)_2_FAPb_2_I_7_ at 0 GPa and 1 GPa

To compare the structural flexibility of the CMA^+^ and PEA^+^ organic cations, first-principle calculations based on DFT were performed to analyze the effects of pressure on the electronic properties of 2D-CMAI and 2D-PEAI perovskites. Figure S8 illustrates the compression of organic layers under a pressure of 0–1 GPa. It is evident from Fig. S8 that CMAI exhibits more significant compression, indicating its flexible characteristics. The structures of 2D-CMAI (*n* = 2, (CMA)_2_FAPb_2_I_7_) and 2D-PEAI (*n* = 2, (PEA)_2_FAPb_2_I_7_) under 0–1 GPa hydrostatic pressure (Fig. 2h) were optimized by releasing the atoms and lattices with the same settings, from which, the lattice parameters, theoretical energy band structures, and density of states (DOS) were calculated. Figure 2c shows that a greater contraction is exhibited in the direction perpendicular to the organic layer (*c*-axis direction shown in Fig. 2h), even though the hydrostatic pressures are applied in all directions. We deduce that this resistance to deformation is conferred by the unique ring breathing vibration mode specific to cyclohexane. It implies that the organic interlayer could act as a spring to relax the pressure. Also, it should be noticed that the compressions of (CMA)_2_FAPb_2_I_7_ along the three directions are considerably milder than those of (PEA)_2_FAPb_2_I_7_ (Fig. 2c), indicating that CMA^+^ could withstand larger strain than that of PEA^+^ under pressure between 0 and 1 GPa.

From the theoretical energy band structure and DOS shown in Figs. 2a, b and S7, the CBMs of both perovskites are found to be primarily contributed by Pb_p orbitals, while I_p orbitals are the main source of the VBM, which are similar to other 2D perovskites in the previous reports [40, 41]. Thus, the electronic characteristics of the 2D perovskite are expected to be affected by the variations in Pb and I under pressure, including both the bond length and bond angles of Pb-I (Fig. 2g). According to the previous research [41–45], the contraction of the metal–halide bond would enhance the orbital overlap, which increases the electronic band dispersion and reduces the bandgap. A decrease in the metal–halide bond angle results in reduced orbital overlap and a wider bandgap. Therefore, competition between these two effects would be generated because both decreased bond angles and bond lengths are expected to occur as pressure rises, leading to a succession of shifts in the band. According to the calculations in Fig. 2d and e, (PEA)_2_FAPb_2_I_7_ experiences significant contraction in Pb-I bond length and a decreased Pb-I-Pb angle, which would contribute to narrowing and widening the bandgaps, respectively. In contrast, the variations in both the Pb-I bond length and Pb-I-Pb angle of (CMA)_2_FAPb_2_I_7_ under pressure are less significant. Therefore, (CMA)_2_FAPb_2_I_7_ exhibits smaller bandgap changes than that of (PEA)_2_FAPb_2_I_7_ under pressure from 0 to 1 GPa, as illustrated in Fig. 2f. To validate the band structure results of the theoretical calculations, we conducted experimental measurements of the UV–Vis absorption spectra for two types of 2D perovskites to determine the bandgap. The experimental results show that (Fig. S9), under conditions of 0–3.1 GPa, (CMA)_2_FAPb_2_I_7_ exhibits smaller bandgap changes, consistent with the theoretical calculations. It is worth noting that in the tests, it was found that this bandgap change with pressure is reversible, meaning that f-PSCs can maintain better structural stability in stress-changing environments, such as repeated bending.

### High Pressure and Carrier Dynamics Characterization of Perovskite

**Fig. 3:**
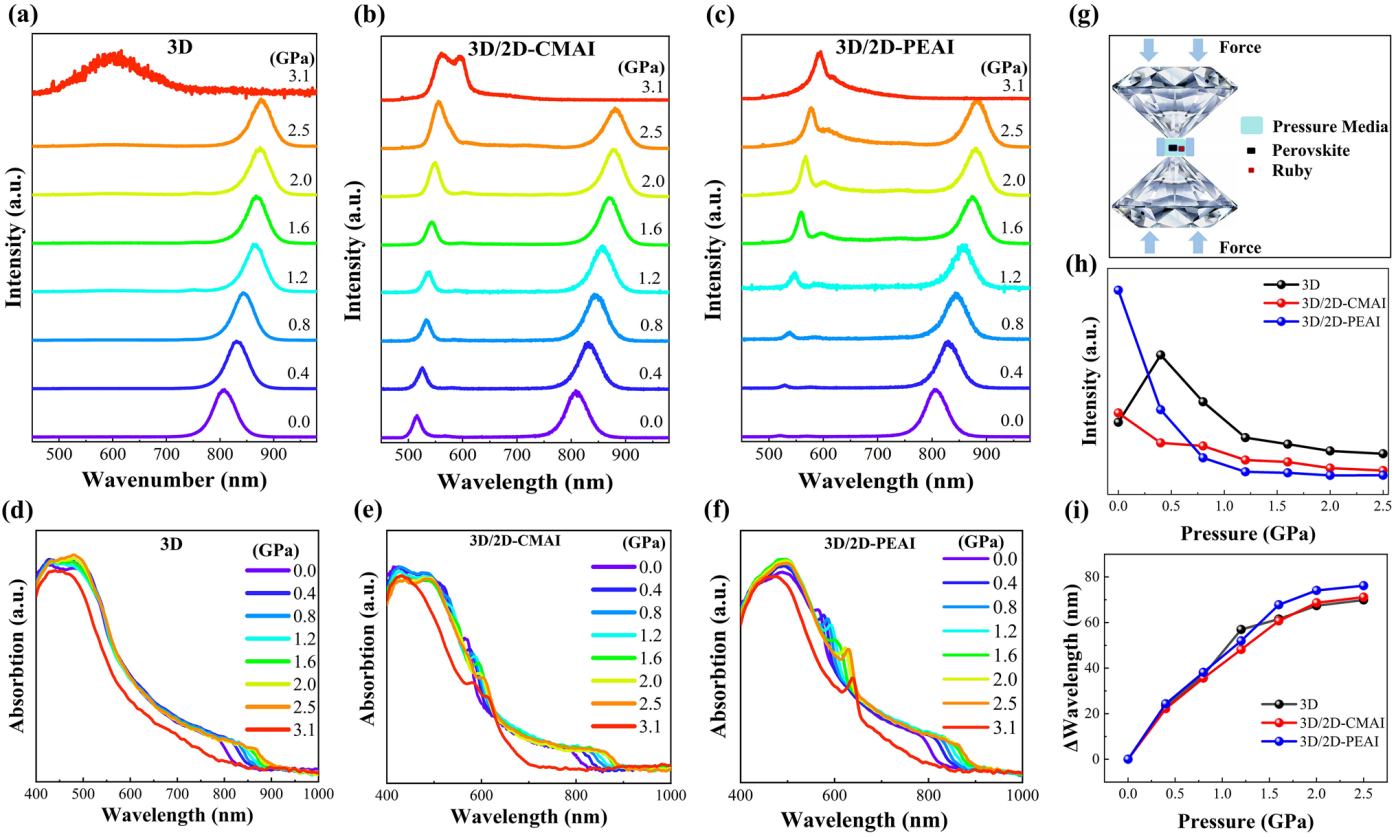
**a–c** PL spectra at selected pressures of pristine 3D, 3D/2D-CMAI, and 3D/2D-PEAI. **d–f** Absorption spectra at different pressures with 3D, 3D/2D-CMAI, and 3D/2D-PEAI. **g** Schematic diagram of DAC pressure device. **h–i** PL intensity and wavelength as a function of pressure

To experimentally evaluate the effects of pressure on the optoelectronic properties of these perovskites, in situ steady-state PL spectra and UV–Vis absorption spectra were obtained using a diamond anvil cell (DAC), as schematically shown in Fig. 3g, where the pressure was gradually increased from 0 to 3.1 GPa. As shown in Fig. 3a, the pristine 3D perovskite exhibits a gradually red-shifted PL peak from 805 to 876 nm with increasing pressure from 0 to 2.5 GPa, which is consistent with the absorption band-edge shifts shown in Fig. 3d. According to our calculation analysis in Fig. 2, this could be explained by the compressed Pb-I bond length under pressure, contributing to the narrower bandgap. When the pressure is further increased to 3.1 GPa, the perovskite crystal undergoes amorphization [46–48]. Upon the incorporation of 2D perovskites, the 3D/2D-CMAI and 3D/2D-PEAI films show additional peaks at 516 and 521 nm, assigned to the (CMA)_2_FAPb_2_I_7_ and (PEA)_2_FAPb_2_I_7_ 2D perovskites, respectively, which also conform to the excitonic absorption peaks shown in Fig. 3e and f. With the structural flexible (CMA)_2_FAPb_2_I_7_ 2D phases acting as a cushion to relax the exerted pressure, the 3D/2D-CMAI film demonstrates milder variations in PL and absorption spectra, including the PL intensity and wavelength changes shown in Fig. 3h and i. Contrastingly, the 3D/2D-PEAI reveals more obvious optoelectronic variations under pressures, due to the structural rigidity of the PEA^+^ organic interlayer. The results are highly consistent with the calculation analysis.

**Fig. 4:**
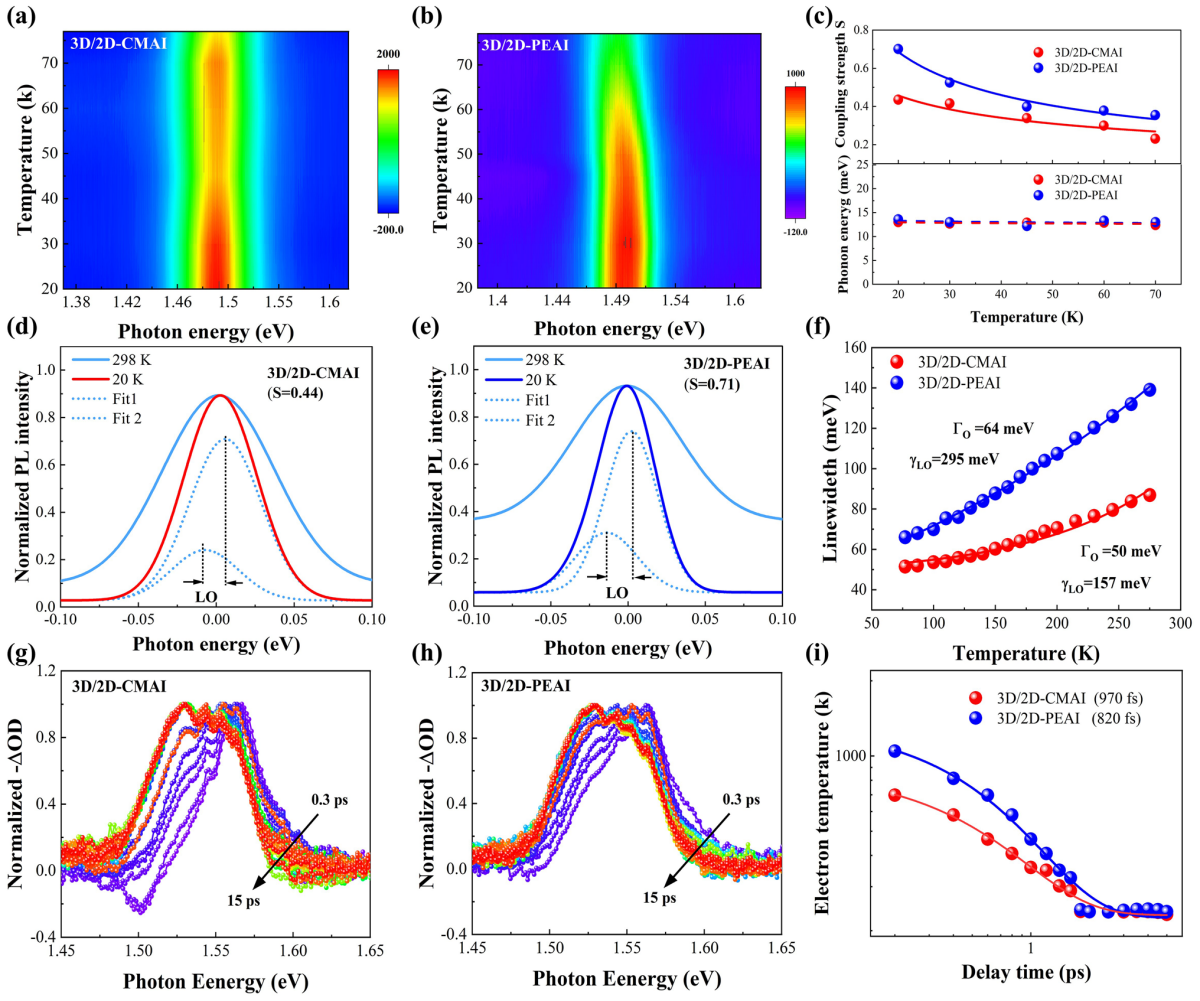
**a, b** Pseudo-color maps of temperature-dependent PL spectra of 3D/2D-CMAI and 3D/2D-PEAI from 20 to 77 K. **c** Derived carrier–LO phonon coupling strength as a function of temperature ranging from 20 to 77 K for 3D/2D-CMAI and 3D/2D-PEAI (top), phonon energies derived from the peak fitting to the PL emission spectra for 3D/2D-CMAI and 3D/2D-PEAI (bottom). **d, e** PL emission spectra of 3D/2D-CMAI and 3D/2D-PEAI measured at 20 and 298 K. **f** Temperature dependence of PL emission spectral width (FWHM) of 3D/2D-CMAI and 3D/2D-PEAI from 77 to 300 K. **g, h** HCs at delay times from 0.3 to 15 ps. **i** Hot electron temperature decay for 3D/2D-CMAI and 3D/2D-PEAI films

To elucidate the impact of different 2D perovskite material rigidity on the optoelectronic performance of PSCs, we conducted cryogenic PL spectroscopic measurements and Femtosecond transient absorption (TAS) spectroscopic measurements to analyze the hot carrier (HC) relaxation processes. The electron–phonon coupling (EPC) process has an important impact on the relaxation of hot carriers. It has been revealed that the relaxation is mostly mediated by the electron–phonon coupling interaction, in which hot carriers relax to band states mainly via emission of phonons [49, 50]. Therefore, inhibiting the electron–phonon coupling can reduce longitudinal optical (LO) phonon emission, thus slowing down the cooling of hot carriers and inhibiting non-radiative recombination. Consequently, understanding the electron–phonon coupling in nanoscale perovskite materials is crucial for controlling the hot carrier cooling process. Iaru et al. examined the Fröhlich interaction-induced coupling of excitons and LO phonons in perovskite, and offered a way to quantify the strength of the Fröhlich interaction using cryogenic PL spectra [51]. The previous research reveals that during the HC cooling process, excess energy is dissipated through the electrostatic interaction between the charge carrier and the lattice oscillations [52], resulting in the emission of phonons with energy lower than that of the zero-phonon band. Based on these principles, the dependence of carrier–LO phonon coupling strength (S) on the dielectric constants of a material can be summarized by the equation: $S=\frac{{e}^{2}}{\hslash c}\sqrt{\frac{{m}^{*}c}{2\hslash {\omega }_{LO}}\left(\frac{1}{{\varepsilon }_{\infty }}-\frac{1}{{\varepsilon }_{0}}\right)}$, where m* is the effective mass of the electron, and *Ɛ*_*0*_ and *Ɛ*_*∞*_ are the high-frequency and static dielectric constants, respectively. In polar materials such as perovskite, where the phonon-assisted process is dominated by the LO phonons, phonon sidebands could be utilized to analyze the interactions between carriers and phonons. Particularly, S could be directly quantified by the PL intensity ratio between the 1st-order LO phonon emission and the zero-phonon band emission [53–55]. PL spectra of the 3D/2D perovskite samples were acquired at cryogenic temperatures to eliminate the broadening of PL peaks caused by thermal contributions and acoustic line broadening. This approach enabled the direct observation of Fröhlich interactions in perovskite. The PL spectra of 3D/2D-CAMI and 3D/2D-PEAI at low temperatures ranging from 20 to 70 K are depicted in Figs. 4a, b and S10. PL intensities gradually increase at lower temperatures, which could be explained by the suppressed non-radiative recombination induced by the defects, due to the lack of thermal stimulation. By comparing the low-temperature PL spectra at 20 K with those at room temperature, it is evident that the 3D/2D-PEAI (Fig. 4e) exhibits a more pronounced low-energy sideband in the PL spectra compared to the 3D/2D-CMAI (Fig. 4d). This difference could be attributed to the stronger exciton–phonon coupling, leading to greater dissipation of energy. The low-energy tail becomes less significant as the temperature increases, and eventually disappears at room temperature. The strength of carrier–phonon coupling can be determined by fitting the asymmetric PL profiles with Gaussian functions at low temperatures, as illustrated in Fig. 4d and e. As depicted in the temperature-dependent S summarized in Fig. 4c, the 3D/2D-CMAI exhibits an overall lower S than that of 3D/2D-PEAI between 20 and 70 K, indicating a significant weakening of Fröhlich interactions between the carrier and LO phonon with the assistance of 2D-CMAI. Similar LO phonon energies (~ 13 meV) between the zero-phonon band and the lower-energy side band are noticeable in Fig. 4c, consistent with the reported LO phonon energy for perovskite [53]. The presence of a low-energy sideband solely originating from the 1st-order LO phonon emission confirms the accuracy of the measurements.

The LO phonon coupling at higher temperatures is also evaluated by the broadening of PL spectra from 77 to 293 K based on the Bose–Einstein thermal distribution $\Gamma (T)={\Gamma }_{0}+{\Gamma }_{LO}(T)={\Gamma }_{0}+\frac{{\Upsilon }_{LO}}{{e}^{{E}_{LO}/kT}-1}$, where Γ_0_ represents the inhomogeneous broadening unrelated to the temperature, while Γ_LO_ indicates the homogeneous broadening originated from the interaction of optical phonons and excitons [56, 57]. As shown in Fig. 4f, the 3D/2D-PEAI shows stronger coupling strength (*γ*_LO_ = 295 meV) than that of 2D-CMAI (*γ*_LO_ = 157 meV). The effects of 2D perovskite on carrier–phonon coupling could be explained by the mechanical properties of the bulky organic cations. The CMA^+^ cation, with superior structural flexibility, could act as a damper to relax the mechanical stresses caused by the lattice oscillations, as supported by the previous pressure-dependent calculations and experimental results.

Further evidence of the weakened electron–phonon coupling strength is demonstrated through the investigation of HC cooling in perovskite using TAS measurements. The bandgap excitation (400 nm pump laser) was used to generate the HC populations in perovskite films. Figure S11 displays the TAS spectral features of perovskite films following excitation at 3.1 eV with an incident power of 30 μW. Figures 4g, h and S12a display the normalized TAS of 3D/2D-CMAI, 3D/2D-PEAI, and 3D films, respectively, within the first 15 ps, extracted from the pseudo-color TAS plots. The dominant ground-state bleach (GSB) band at 1.54 eV appears due to the band-filling effect in the three films. After excitation, the initial non-equilibrium HC would rapidly undergo a scattering process and cool down to a Fermi–Dirac distribution. The high-energy tail of the GSB observed in Fig. 4g and h represents the Fermi–Dirac distribution, whose gradual narrowing could be attributed to the HC cooling. The photo-induced absorption (PIA) band near 1.60 eV observed within a short delay time (< 3 ps) could be assigned to the bandgap renormalization caused by HC thermalization. The balance between the red-shift caused by bandgap renormalization and the blue-shift caused by the Burstein–Moss effect results in a slight red-shift in the GSB peak. By fitting the high-energy tail using the Maxwell–Boltzmann distribution function [28, 58]: $\frac{\Delta T}{T}\left(E\right)\sim {\text{exp}}\left(-\frac{E-{E}_{{\text{f}}}}{{{k}_{{\text{B}}}T}_{{\text{C}}}}\right)$, where *k*_B_ denotes the Boltzmann constant and the Fermi energy (*E*_f_), the effective carrier temperature *T*_C_ can be extracted as a function of delay time. *T*_C_ of three perovskite films was analyzed and compared, as depicted in Figs. 4i and S12b. The bi-exponential fitting of the data yields the following HC cooling times of 460, 970, and 820 fs for 3D, 3D/2D-CMAI, and 3D/2D-PEAI films, respectively. The remarkably prolonged relaxation lifetime of the HC in the 3D/2D-CMAI film is attributed to the weaker Fröhlich coupling strength between the LO phonons and carriers [53], which, in turn, validates the cryogenic PL results. To further study the HC dynamics, the TAS of three perovskite samples were recorded under bandgap (1.55 eV) excitation. The samples exhibited GSB at 805 nm, similar to those observed under excitation of 3.1 eV. While disparate from the previous scenario in Fig. 4g and h, the TAS of perovskite samples in Fig. S13 shows the absence of high-energy tails and a PIA peak at the short delay time, implying the insufficient number of HC formed under bandgap excitation. Thus, excitation energy much larger than the perovskite bandgap should be a prerequisite for stimulating the HC. The advanced HC cooling could also help to reduce the thermalization losses in PSCs.

To investigate the chemical passivation effects of 2D phases on FAPbI_3_ 3D perovskite, XPS measurements were conducted to analyze the surface chemical composition of 3D and 3D/2D perovskite films. The binding energy of each element was analyzed after correcting the carbon spectrum. The high-resolution XPS spectra of Pb, I, and N are displayed in Fig. S14. It is observed that both 3D/2D-CMAI and 3D/2D-PEAI exhibit XPS spectra shifts of Pb 4*f*, I 3*d*, and N 1*s* by 0.1–0.2 eV toward higher binding energies, respectively, compared to those of 3D perovskite. The shifts of both I 3*d* and N 1*s* toward higher binding energies could be attributed to the stronger N–H···I bond between CMA^+^/PEA^+^ and FAPbI_3_. The shift of the Pb 4*f*_5/2_ and Pb 4*f*_7/2_ to higher binding energies may be assigned to the interactions between uncoordinated Pb with CMA^+^/PEA^+^. Both effects could effectively passivate the Pb and I vacancies in FAPbI_3_, thereby preventing non-radiative recombination losses in the PSCs. The charge dynamics of the three perovskite samples under light illumination were then examined using PL and time-resolved PL (TRPL) measurements. The inclusion of PEAI and CMAI has been found to increase the PL emission in a stepwise manner. Similarly, when fitting the TRPL spectra in Fig. S15 with a bi-exponential function yields carrier lifetimes of 0.96, 1.84, and 1.74 μs for 3D, 3D/2D-CMAI, and 3D/2D-PEAI perovskite films, respectively (Table S1). This suggests that the 2D-PEAI and 2D-CMAI phases passivate non-radiative recombination sites [59–62]. It is evident that 2D-CMAI more effectively suppresses the non-radiative recombination rates and losses in 3D perovskite, despite the similar chemical passivation effects of the two 2D perovskites, as indicated by XPS spectra. This could be explained by weaker electron coupling and the resulting milder displacement of the surrounding organic octahedral structure following electron capture, which could alleviate the non-radiative electron capture rate. Such a conclusion would be further supported by device characterizations in the subsequent context.

### Performance and Stability of the Photovoltaic Devices

**Fig. 5:**
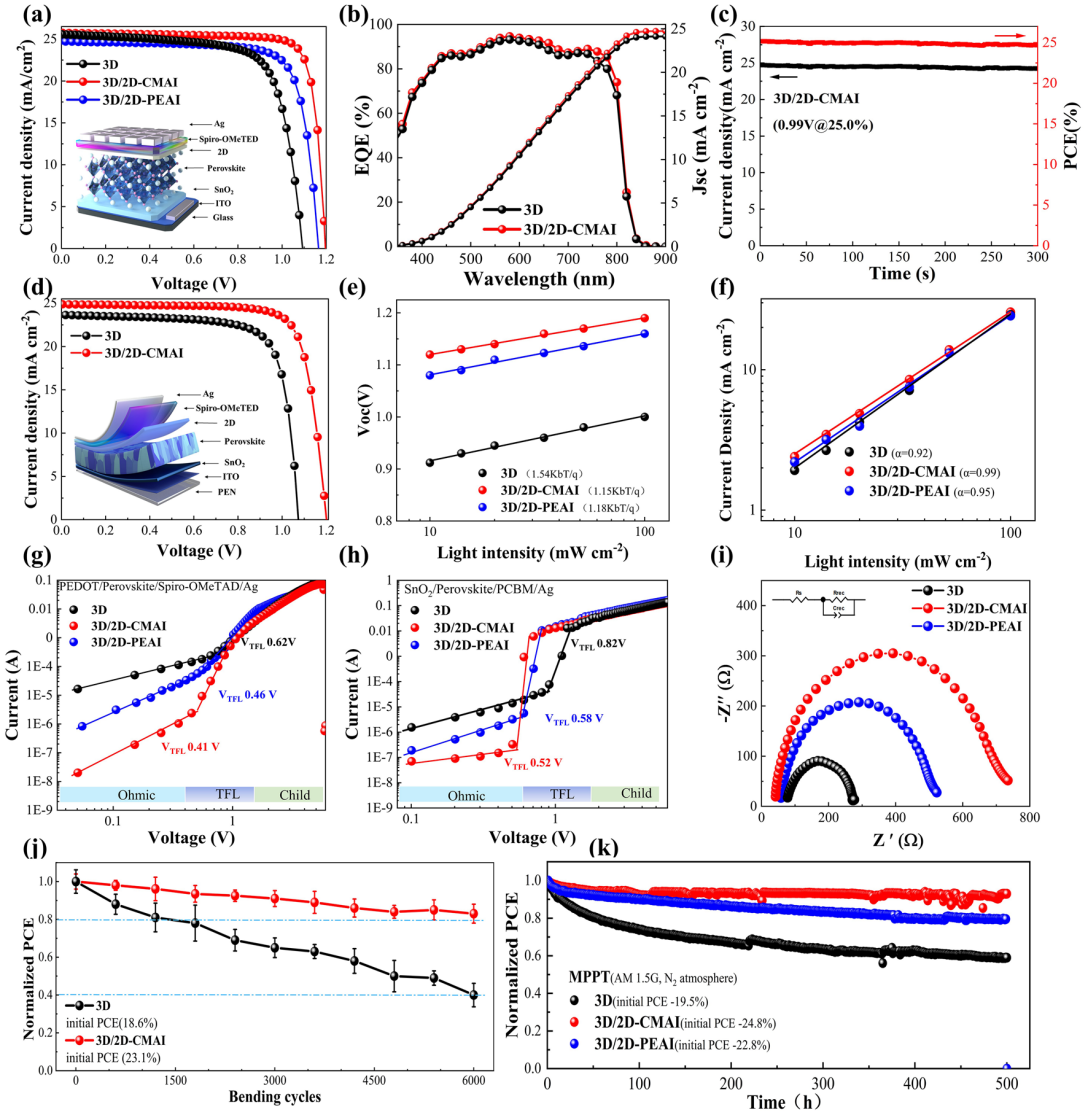
**a** *J–V* characteristics of optimized PSCs and the corresponding schematic illustrating of rigid PSCs (inset).** b** EQE spectra and the integrated current density. **c** Stabilized power output (SPO) at maximum power point tracking under working conditions with 100 mW cm^−2^ irradiation. **d** *J–V* characteristics of optimized f-PSCs and the corresponding schematic illustration of f-PSCs (inset). **e, f** Light intensity-dependent *V*_OC_ and *J*_SC_.** g, h** Trap concentration estimated by dark *J–V* curves. **i** Electrochemical impedance spectroscopy of PSCs and the corresponding equivalent circuit model (inset). **j** Mechanical test of the f-PSCs based on bending radius of 5 mm. **k** Maximum power point tracking (MPPT) of 3D devices and 3D/2D-CMAI, 3D/2D-PEAI devices under 1 sun illumination in the N_2_ environment

To evaluate the effect of organic cations with different structural rigidities on the photovoltaic performance of PSCs, PSCs with the device architecture of ITO/SnO_2_/3D/2D/Spiro-OMeTAD/Ag were constructed and tested under simulated 1 sun illumination at an intensity of 100 mW cm^−2^ (AM 1.5 spectrum). The details of the deposition process are described in the experimental section. The optimal concentrations of CMAI and PEAI are first determined from Tables S2 and S3. The current density–voltage (*J*–*V*) characteristics of the leading devices are shown in Fig. 5a, and the photovoltaic performances are summarized in Table S4. The control device shows PCE of 20.2% with short-circuit current (*J*_SC_) of 25.4 mA cm^−2^, open-circuit voltage (*V*_OC_) of 1.09 V, and fill factor (*FF*) of 72.38%. With the incorporation of the 2D-PEAI phase, the PCE exhibits an improvement of 22.4%, which can be attributed to the passivation effects of the 2D-PEAI as discussed previously. Therefore, a significantly improved *V*_OC_ (1.16 V) is observed due to the suppressed non-radiative recombination losses, while a slightly decreased *J*_SC_ (24.69 mA cm^−2^) might be attributed to the inhibited carrier transport by the bulky organic spacer. For 3D/2D-CMAI-based PSCs, the PCE is further increased to 25.5%, with simultaneously improved *V*_OC_ to 1.19 V, *J*_SC_ to 25.73 mA cm^−2^, and *FF* to 83.09%. This achievement stands out as one of the highest reported PCEs to date. Figure S16 and Table S5 show the forward and reverse scan *J*–*V* characteristics of the champion PSC based on 3D and 3D/2D-CMAI, which exhibits a negligible 0.67% hysteresis index in 3D/2D-CMAI, but increases to 2.6% in 3D. Three-dimensional/2D-CMAI significantly reduces the hysteresis. The EQE of PSCs based on 3D and 3D/2D-CMAI are compared in Fig. 5b. The integration of these efficiencies provides the *J*_SC_ of 24.45 and 24.73 mA cm^−2^, respectively, consistent with the *J*–*V* characteristics. Based on the previous characterizations, the presence of structurally flexible CMA^+^ abates the carrier and phonon coupling strength, which might decrease the associated electron capture by the defect states, in addition to chemical passivation on the halide or lead vacancies, thus increasing the defect tolerance of perovskite. It ultimately minimizes the SRH losses and gives rise to an exceptional *V*_OC_ value of 1.20 V (the highest *V*_OC_ in this work as shown in Table S2). We observed that this is one of the highest *V*_OC_ records achieved for narrow bandgap FAPbI_3_ perovskite to date. The device was then tracked at the maximum power point for 300 s, and the stabilized power output is presented to be 25.0% (Fig. 5c), which aligns well with the *J*–*V* characteristics.

Furthermore, the soft nature of CMA^+^ makes it a great candidate for the development of f-PSCs based on a 3D/2D system. Figure S17 assesses the mechanical properties of the perovskite films with and without 2D phases using peak force quantitative nano-mechanical mapping. The averaged Derjaguin–Muller–Toporov (DMT) modulus of the 3D/2D-CMAI film is observed to be 5.14 GPa, which is lower than that of the pristine 3D film (8.81 GPa) and the 3D/2D-PEAI film (7.68 GPa). This suggests a higher mechanical flexibility of the 3D/2D-CMAI film. We attribute this to the fact that the 2D layer formed by soft CMAI could provide perfect mitigation for mechanical stress. The *J*–*V* characteristics of f-PSCs based on 3D/2D-CMAI are, therefore, studied in Fig. 5d, in comparison with those of the control device. From the photovoltaic performances summarized in Table S6, the 3D/2D-CMAI-based f-PSCs demonstrate a notably increased PCE from 19.3% (3D-based f-PSCs) to 23.4%, with *V*_OC_ of 1.20 V,* J*_SC_ of 24.87 mA cm^−2^, and *FF* of 78.39%, as shown in Table S7. The EQE of f-PSCs based on 3D/2D-CMAI are illustrated in Fig. S18, the integration of which provides the *J*_SC_ of 24.34 mA cm^−2^, consistent with the *J*–*V* characteristics. The superior device performance could be accredited to the improved mechanical modulus of the 3D/2D film with a soft CMA^+^ organic spacer, which also results in exceptional mechanical flexibility by maintaining 83% of the initial PCE after 6000 bending cycles (with a bending radius of 5 mm), as illustrated in Fig. 5j. In contrast, the pristine 3D-based PSCs display only 41% of the original PCE. The result perfectly illustrates the softness of the CMA^+^ interlayer for improving the interfacial characteristics of the 3D/2D hetero-structure.

The *V*_OC_/*J*_SC_ as a function of light intensity was investigated to elucidate the recombination mechanism in PSCs based on 3D and 3D/2D perovskite systems. The relationship between V_OC_ and light intensity is assessed in Fig. 5e and f using the equation ${V}_{{\text{OC}}}\propto \left(n{K}_{{\text{B}}}T/q\right){\text{ln}}\left({P}_{{\text{light}}}\right)$, where *n*, *k*_B_, *T*, and *q* are the constants representing the ideality factor, Boltzmann constant, temperature, and elementary charge, respectively. The deviations of the *n* value from 1 imply the presence of trap-assisted recombination in the PSCs. The decrease in the n value from 1.54 for pristine PSCs to 1.15 and 1.18 for 3D/2D-CMAI and 3D/2D-PEAI modified PSCs, respectively, suggests the suppression of trap-assisted non-radiative recombination in the devices. In addition, based on $\mathrm{\alpha }={\text{ln}}\left({J}_{{\text{SC}}}\right)/{\text{ln}}\left({P}_{{\text{light}}}\right)$, the slope values α for the devices 3D, 3D/2D-CMAI, and 3D/2D-PEAI can be calculated to be 0.92, 0.99, and 0.95, respectively. The decrement in α indicates less recombination in devices with 2D incorporation. The trap density of the perovskite films is subsequently assessed by the space charge-limited current model based on the equation: ${n}_{{\text{trap}}}=\frac{{2\varepsilon }_{0}\varepsilon {V}_{{\text{TFL}}}}{{{\text{eL}}}^{2}}$, where *ε*_0_ is the vacuum dielectric constant, *e* is the charge of the electron, ε is the dielectric constant, *L* is the perovskite layer thickness, and *V*_TFL_ is the trap filling voltage. The thicknesses of the 3D, 3D/2D-CMAI, and 3D/2D-PEAI films were determined to be 500, 565, and 515 nm, respectively (Fig. S19).

The dark current–voltage curves of electron-only devices (ITO/SnO_2_/perovskite/PCBM/Ag) and hole-only devices (ITO/PEDOT:PSS/perovskite/spiro-OMeTAD/Ag) are presented in Fig. 5g and h. These curves were used to determine the electron and hole trap densities of the 3D and 3D/2D films, and the findings are summarized in Tables S8 and S9. It can be observed that the incorporation of PEAI and CMAI leads to a decrease in both electron and hole trap densities. This may be attributed to the improved perovskite crystallization and passivation effects of the 2D phases. Additionally, the similar extracted ${{\text{n}}}_{{\text{trap}}}$ values for both 3D/2D-CMAI and 3D/2D-PEAI perovskites imply comparable chemical passivation effects of 2D-CMAI and 2D-PEAI, consistent with the previous XPS results. In addition, the charge mobilities of perovskite films were evaluated by the equation: $J=\frac{9}{8}{\varepsilon }_{0}\varepsilon {\mu }_{h}\frac{({{V}_{a}-{V}_{bi})}^{2}}{{L}^{3}}$. The calculated results of the hole and electron mobilities are also shown in Tables S9 and S10. The improved electron and hole mobilities of the 2D/3D hybrid film may indicate the preferred orientation of perovskite crystals with 2D assistance, thereby promoting the charge transport properties. While analyzing the Nyquist plots of the PSCs under illumination (Fig. 5i), a significantly larger recombination resistances (*R*_rec_) is observed in the 3D/2D-CMAI (582 Ω) device compared to the 3D/2D-PEAI (341 Ω) device. This indicates a reduction in non-radiative recombination with the incorporation of 2D-CMAI. These results confirm a clear correlation between the electron–phonon coupling and the non-radiative recombination process. The reduced non-radiative decay may be explained by the fact that in 3D/2D-CMAI hybrid systems with weaker electron–phonon coupling, more low-frequency phonons are expected to couple with photo-generated carriers. It would thus reduce the non-adiabatic coupling, which slows down the electron–hole non-radiative recombination rate and increases the defect tolerance of the perovskite film.

Despite the high PCEs achieved by FAPbI_3_-based PSCs, their practical application is still considered a great challenge due to the poor stability of the black α-FAPbI_3_ phase. Previous characterizations have shown that the incorporation of 2D-CMAI phases can effectively alleviate the residual tensile stress within the FAPbI_3_ lattice and thin film. This is expected to improve the intrinsic phase stability of FAPbI_3_-based PSCs. Figure 5k compares the operational stability of 3D and 3D/2D-CMAI, 3D/2D-PEAI PSCs, which was tested by tracking the maximum power point (MPP) under 1 sun illumination in the N_2_ environment. The 3D/2D-CMAI PSCs demonstrate significantly improved operational stability with over 91% of the initial PCE maintained after 500 h of continuous illumination, 3D/2D-PEAI retained 80% of its initial PCE, while that of the 3D device shows rapid degradation with only 60% of the original PCE retained. The encapsulated devices are used for advanced testing, targeting the advanced dark storage test scheme for ISOS-D (International Summit on Organic Photovoltaic Stability dark, at approximately 85% relative humidity and approximately 65 °C temperature). From Fig. S20, it can be seen that 3D/2D-CMAI PSCs maintained 79% initial PCE after 400 h of aging, while 3D/2D-PEAI maintained 67% initial efficiency, which is in sharp contrast to the rapid degradation of 3D-PSCs. The stronger resilience of 3D/2D-CMAI PSCs to the hot-moisture test reflects the improved intrinsic structural stability of the CMAI 2D layer in the hot and humid environment, which may result in the long-term stability of the target perovskite thin-film's crystal structure.

## Conclusions

In conclusion, the CMA^+^ spacer has been incorporated into FAPbI_3_ perovskite to form 3D/2D hetero-structure-based PSCs. The CMA^+^ cation, known for its superior structural softness, has been shown to act as a damper, relaxing the mechanical stress caused by lattice oscillations, in clear contrast to its rigid PEA^+^ analog. It effectively abates the Fröhlich interactions between carriers and LO phonons, which is expected to slow down the carrier capture rate and suppress the non-radiative recombination losses. A V_OC_ record of 1.20 V has been achieved for narrow bandgap perovskite (FAPbI_3_), with an impressive PCE of 25.5%, which is one of the highest reported PCEs for PSCs to date. Additionally, the soft CMA^+^ interlayer enables the fabrication of mechanically robust f-PSCs, resulting in an excellent PCE of 23.4% and good mechanical stability. This work provides a comprehensive understanding of addressing the coupling between electronic charge state and lattice distortion, as well as its correlation with non-radiative recombination losses. It is critically important to develop and screen more effective defect passivators for high-performance PSCs.

## Supplementary Information

Below is the link to the electronic supplementary material.

Supplementary file1 (PDF 1664 kb)
