# Supplementary material for: Structurally Flexible 2D Spacer for Suppressing the Electron–Phonon Coupling Induced Non-Radiative Decay in Perovskite Solar Cells
Source: Nanomicro Lett. 2024 Apr 24;16:178. doi: 10.1007/s40820-024-01401-9 (PMC11043286; doi:10.1007/s40820-024-01401-9)
Supplement: Supplementary file 1 — Supplementary file1 (PDF 1664 kb) [file 40820_2024_1401_MOESM1_ESM.pdf]

Supporting Information for

# Structurally Flexible 2D Spacer for Suppressing the Electron-Phonon Coupling Induced Nonradiative Decay in Perovskite Solar Cells

Ruikun Cao[1,2,#], Kexuan Sun[1,#], Chang Liu[1,*], Yuhong Mao[4], Wei Guo[1], Ping Ouyang[1], Yuanyuan Meng[1], Ruijia Tian[1], Lisha Xie[1], Xujie Lü[4], and Ziyi Ge[1,3,*]

[1] Zhejiang Provincial Engineering Research Center of Energy Optoelectronic Materials and Devices, Ningbo Institute of Materials Technology & Engineering, Chinese Academy of Sciences, Ningbo 315201, P. R. China

[2]School of Materials Science and Chemical Engineering Ningbo University, Ningbo 315211, P. R. China

[3] Center of Materials Science and Optoelectronics Engineering University of Chinese Academy of Sciences, Beijing 100049, P. R. China

[4] Center for High Pressure Science and Technology Advanced Research (HPSTAR), Shanghai 201203, P. R. China

[#]Ruikun Cao and Kexuan Sun contributed equally to this work.

*Corresponding authors. E-mail: liuchang1@nimte.ac.cn (Chang Liu); geziyi@nimte.ac.cn (Ziyi Ge)

## Supplementary Figures

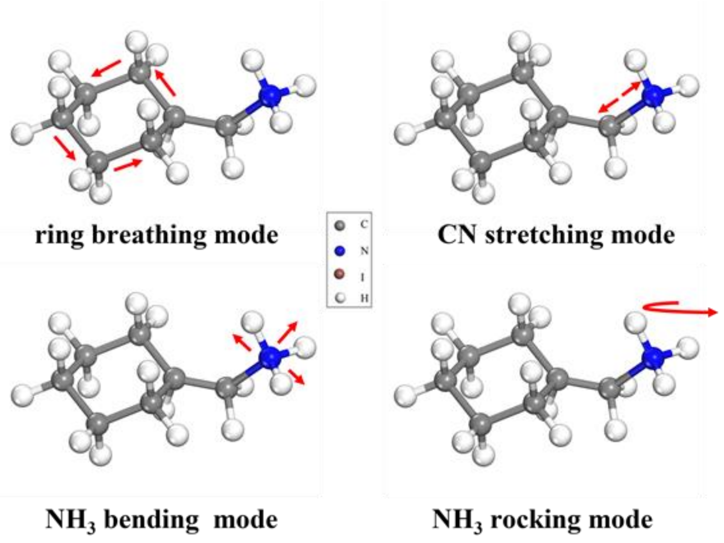


**Fig. S1** Relevant vibrational modes in $CMA^+$

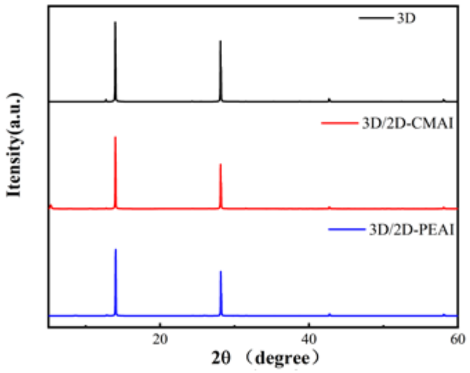


**Fig. S2** X-ray diffraction patterns of 5-60° with 3D, 3D/2D-CMAI and 3D/2D-PEAI films

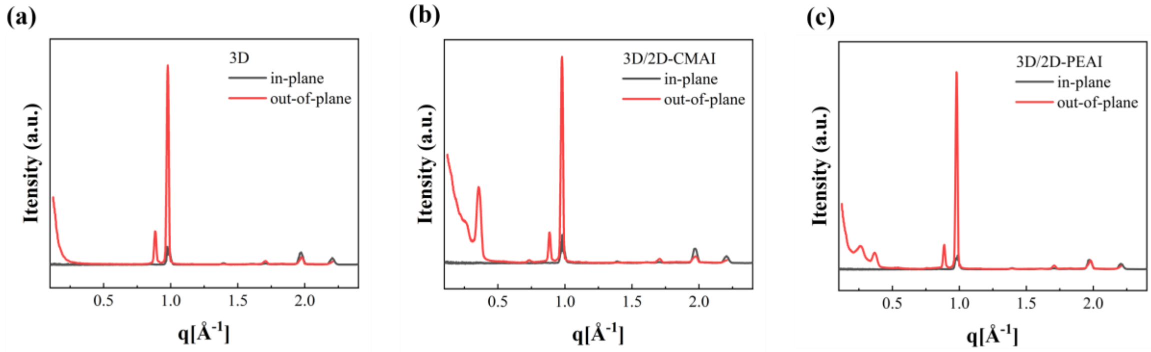


**Fig. S3** The (100) in-plane and out-of-plane line cuts of GIWAX images of 3D, 3D/2D-CMAI, and 3D/2D-PEAI

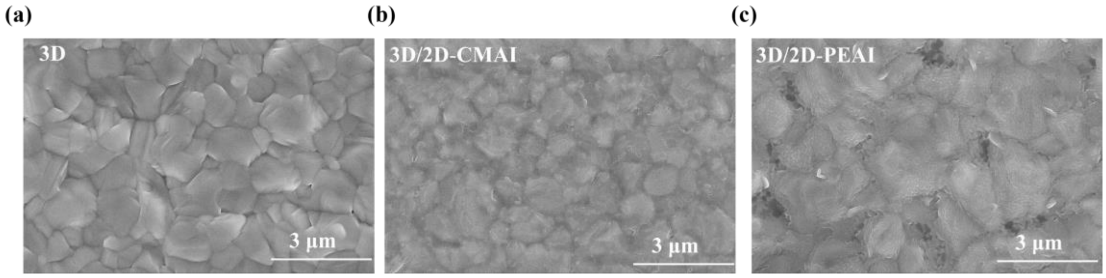


**Fig. S4** SEM images of perovskite surface based on 3D, 3D/2D-CMAI, and 3D/2D-PEAI

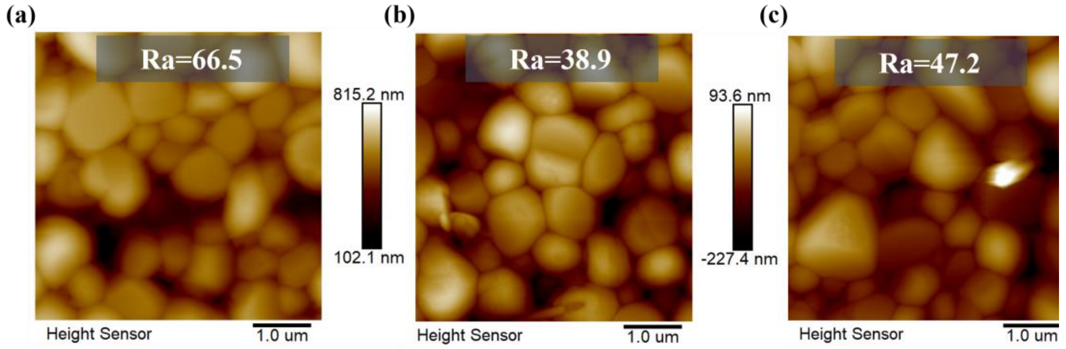


**Fig. S5** AFM images of 3D, 3D/2D-CMAI, and 3D/2D-PEAI

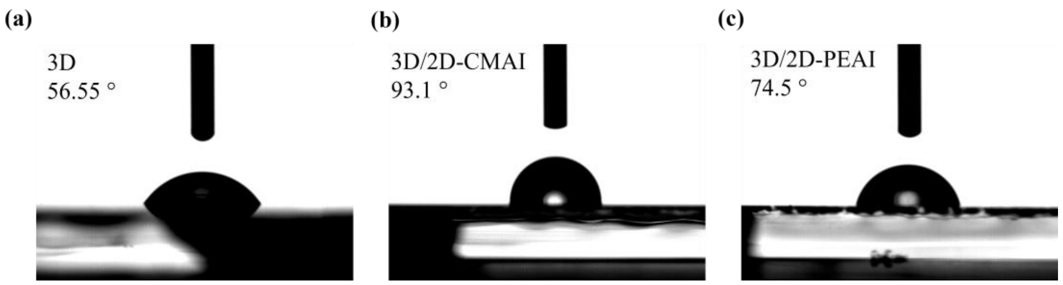


**Fig. S6** Water contact angle test

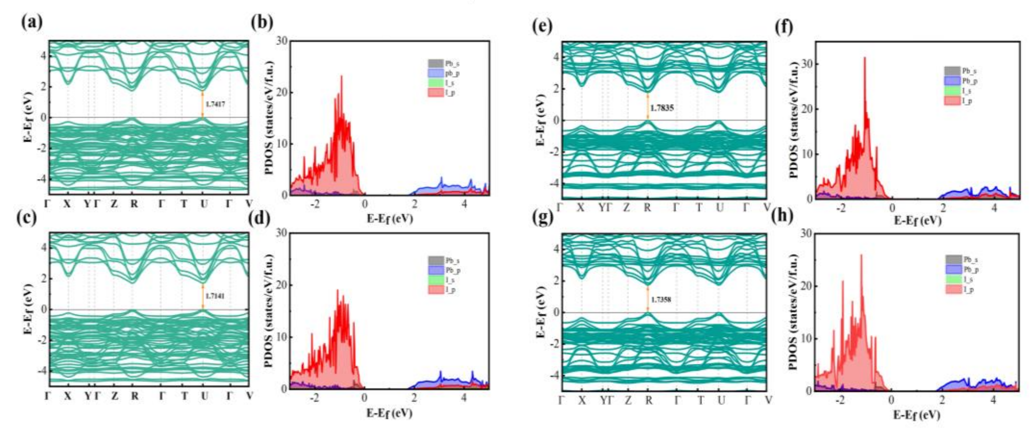


**Fig. S7 a** Theoretical energy band structure of $(CMA)_2FaPb_2I_7$ at 0 GPa. **b** Density of states of $(CMA)_2FaPb_2I_7$ at 0 GPa. **c** Theoretical energy band structure of $(CMA)_2FaPb_2I_7$ at 1 GPa. **d** Density of states of $(CMA)_2FaPb_2I_7$ at 1 GPa. **e** Theoretical energy band structure of $(PEA)_2FaPb_2I_7$ at 0 GPa. **f** Density of states of $(PEA)_2FaPb_2I_7$ at 0 GPa. **g** Theoretical energy band structure of $(PEA)_2FaPb_2I_7$ at 1 GPa. **h** Density of states of $(PEA)_2FaPb_2I_7$ at 1 GPa

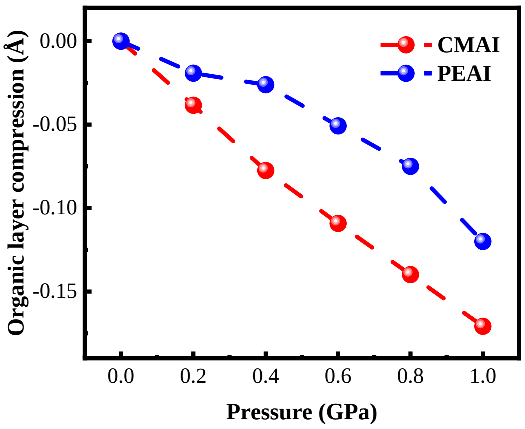


**Fig. S8** Compression diagram of organic layer under 0-1Gpa

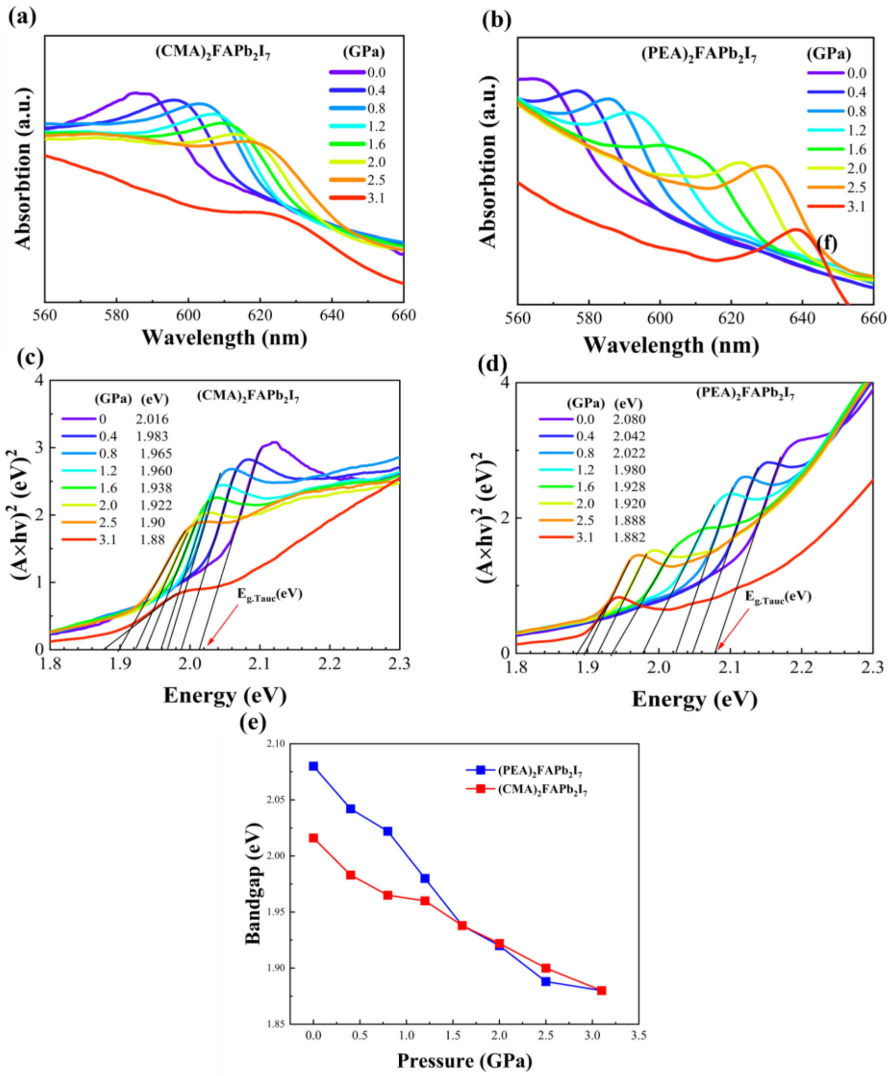


**Fig. S9 a-b** UV-Vis absorption spectra for the ($(CMA)_2FAPb_2I_7$) and ($(PEA)_2FAPb_2I_7$) target perovskite films under 0-3 GPa. **c-d** The corresponding tauc plots of UV-Vis absorption spectra. **e** Variation of the band gap of ($(CMA)_2FAPb_2I_7$) and ($(PEA)_2FAPb_2I_7$) with pressure change

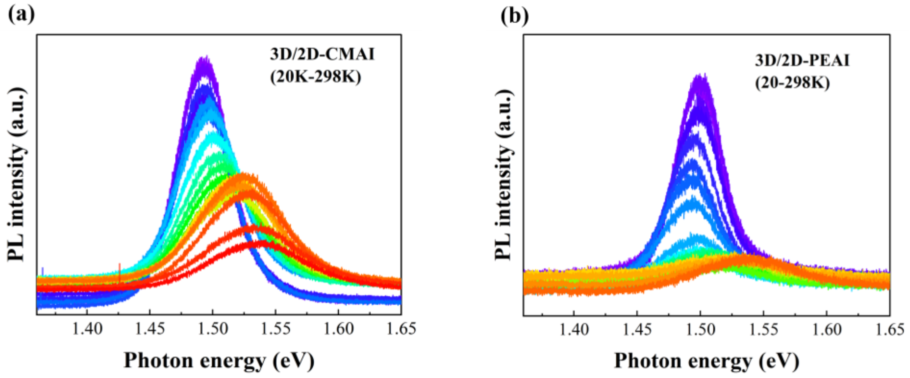


**Fig. S10 a** Low temperature PL testing of 3D/2D-CAMI, (b) Low temperature PL testing of 3D/2D-PEAI

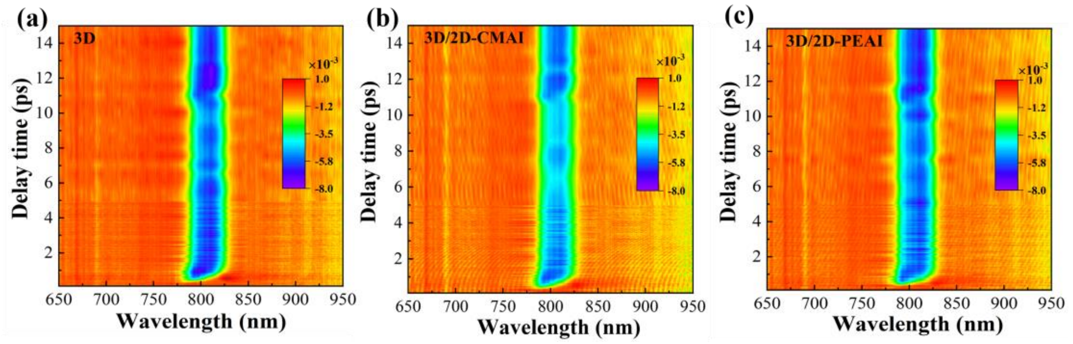


**Fig. S11** Pseudocolor maps of TA spectra for 3D, 3D/2D-CMAI and 3D/2D-PEAI films

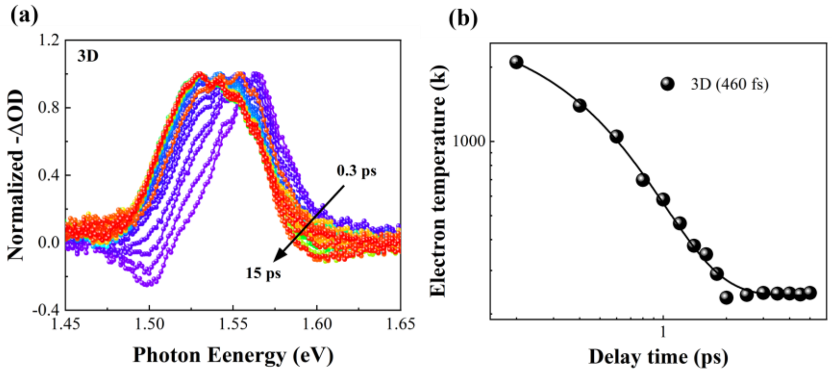


**Fig. S12 a** HCs at delay times from 0.3 to 15 ps for 3D. **b** Hot electron temperature decay for 3D

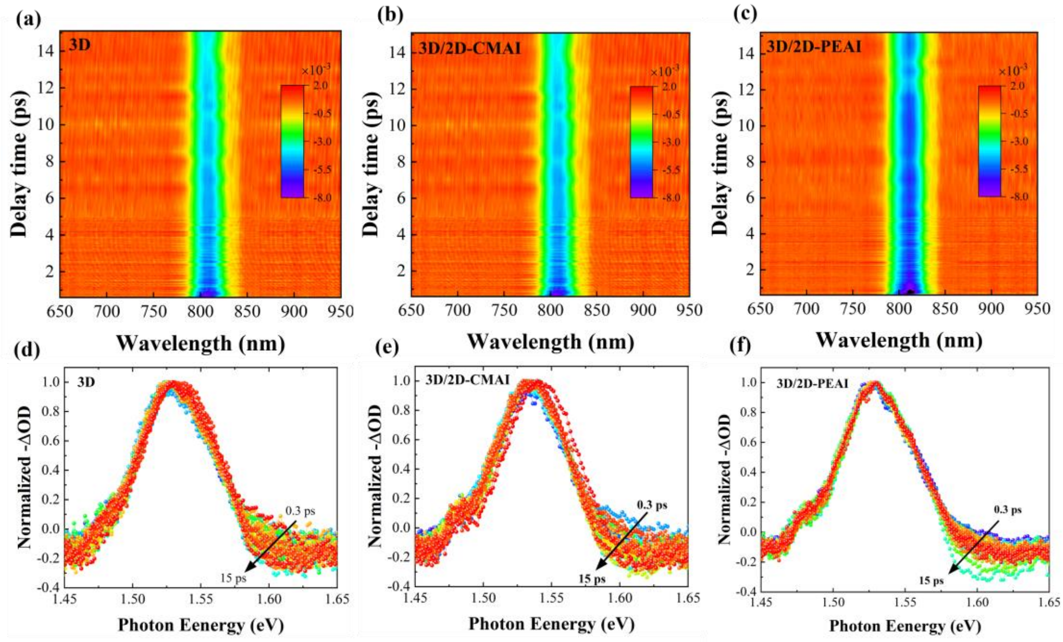


**Fig. S13** Excitation at λ=800 nm **a–c** Pseudocolor maps of TA spectra for pristine 3D and 3D/2D-CMAI,3D/2D-PEAI, **d–f** HCs at delay times from 0.3 to 15 ps

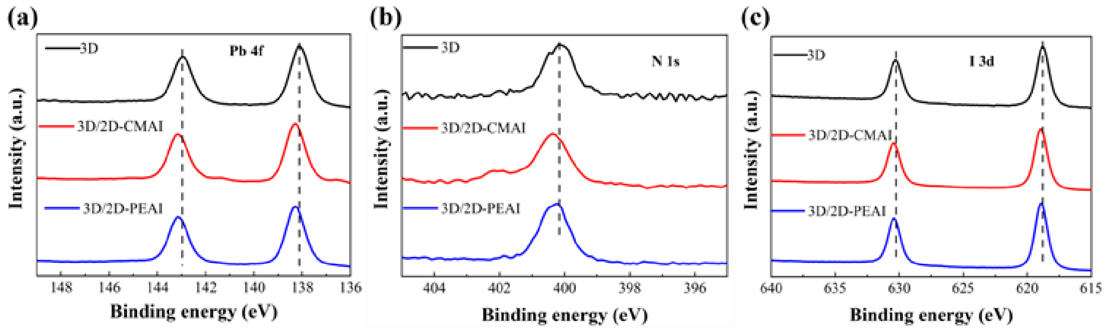


**Fig. S14** High-resolution XPS spectra of Pb 4f, I 3d, and N1s

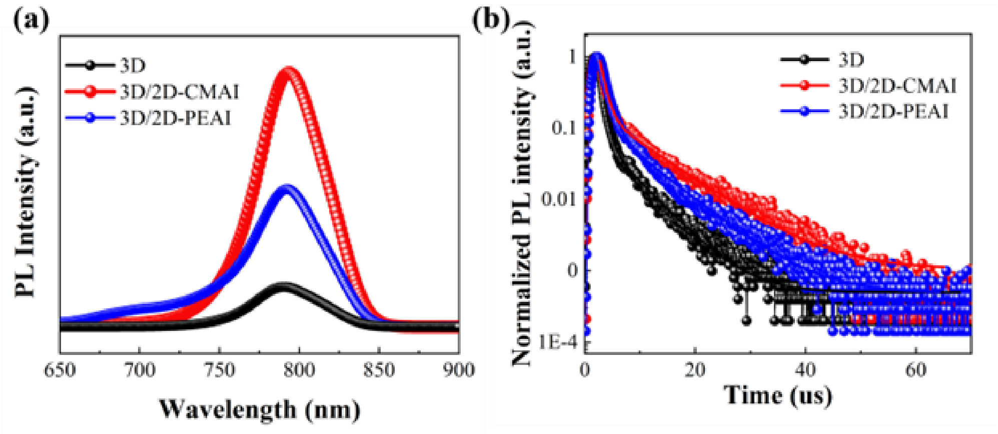


**Fig. S15** Steady-state PL spectra and TRPL spectra of the pristine 3D, 3D/2D-CMAI, and 3D/2D-PEAI

**Table S1** Fitting parameters for TRPL of bi-exponential decay fits

| | $\tau_1$ (μs) | $\tau_2$ (μs) | $\tau$ (μs) |
|---|---|---|---|
| 3D | 0.78 | 5.32 | 0.96 |
| 3D/2D-CMAI | 0.96 | 8.25 | 1.84 |
| 3D/2D-PEAI | 1.17 | 7.11 | 1.74 |

**Table S2** Summary of PSC photovoltaic efficiency based on modification of various concentrations of CMAI

| CMAI | $V_{OC}$ [a] (V) | $J_{SC}$ [a] (mA $cm^{-2}$) | FF [a] (%) | PCE [a] (%) |
|---|---|---|---|---|
| 1.5 mg/ml | 1.17±0.005 | 25.45±0.42 | 79.20±1.03 | 23.71±0.28 |
| | (1.17) | (25.87) | (78.75) | (23.99) |
| 3.0 mg/ml | 1.18±0.006 | 25.53±0.59 | 80.71±1.16 | 24.43±0.40 |
| | (1.19) | (25.46) | (81.88) | (24.83) |
| 5mg/ml | 1.19±0.01 | 25.74±0.26 | 82.11±0.97 | 25.23±0.29 |
| | （1.19） | （25.73） | （83.09） | （25.52） |
| 7 mg/ml | 1.17±0.009 | 25.09±0.33 | 78.93±1.36 | 23.26±0.80 |
| | (1.18) | (25.43) | (79.98) | (24.07) |

[a] Average and standard deviation values were obtained based on over 10 cells from 2 different

**Table S3** Summary of PSC photovoltaic efficiency based on modification of various concentrations of PEAI

| PEAI | $V_{OC}$ [a] (V) | $J_{SC}$ [a] (mA $cm^{-2}$) | FF [a] (%) | PCE [a] (%) |
|---|---|---|---|---|
| 1.5 mg/ml | 1.12±0.038 | 24.76±1.48 | 73.87±3.67 | 20.57±0.45 |
| | (1.15) | (24.79) | (73.12) | (21.01) |
| 3.0 mg/ml | 1.14±0.014 | 24.57±0.40 | 75.59±1.78 | 21.36±0.43 |
| | (1.15) | (24.47) | (77.38) | (21.79) |
| 4.5mg/ml | 1.16±0.018 | 24.39±0.30 | 77.46±0.52 | 22.04±0.40 |
| | (1.16) | (24.69) | (77.98) | (22.45) |
| 6 mg/ml | 1.16±0.011 | 24.20±0.47 | 76.19±1.76 | 21.46±0.69 |
| | (1.16) | (24.61) | (77.44) | (22.15) |

[a] Average and standard deviation values were obtained based on over 10 cells from 2 different batches for 3D/2D-PEAI. Parameters of the best cell are reported in brackets.

**Table S4** Summary of rigid PSC photovoltaic efficiency based on modification with CMAI and PEAI

| Additive | $V_{OC}$ (V) | $J_{SC}$ (mA cm$^{-2}$) | FF (%) | PCE (%) |
|---|---|---|---|---|
| 3D | 1.09 | 25.54 | 72.38 | 20.21 |
| 3D/2D-CMAI | 1.19 | 25.73 | 83.09 | 25.52 |
| 3D/2D-PEAI | 1.16 | 24.69 | 77.98 | 22.45 |

**Table S5** Photovoltaic efficiency of champion PSCs based on 3D and 3D/2D-CMAI in forward and reverse scans

| Additive | | $V_{OC}$(V) | $J_{SC}$ (mA cm$^{-2}$) | FF (%) | PCE (%) |
|---|---|---|---|---|---|
| 3D/2D-CMAI | Reverse | 1.19 | 25.73 | 83.09 | 25.52 |
| | Forward | 1.19 | 25.71 | 82.64 | 25.35 |
| 3D | Reverse | 1.09 | 25.54 | 72.38 | 20.21 |
| | Forward | 1.08 | 24.99 | 72.37 | 19.70 |

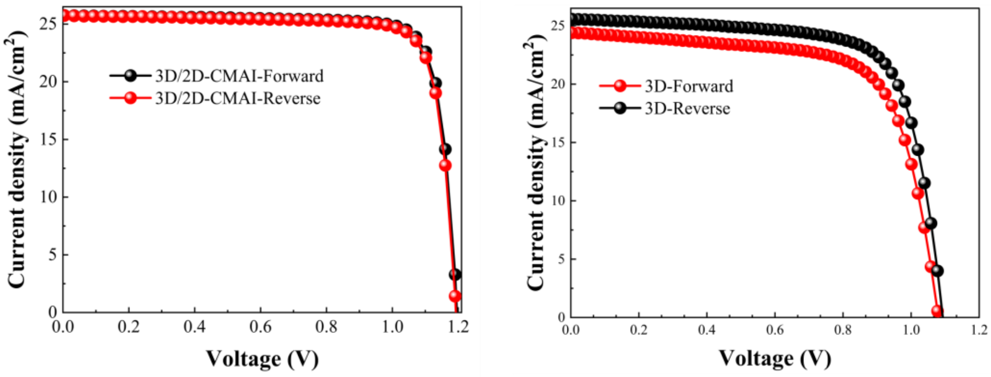


**Fig. S16** J–V characteristics of optimized 3D and 3D/2D-CMAI PSCs in forward and reverse scans

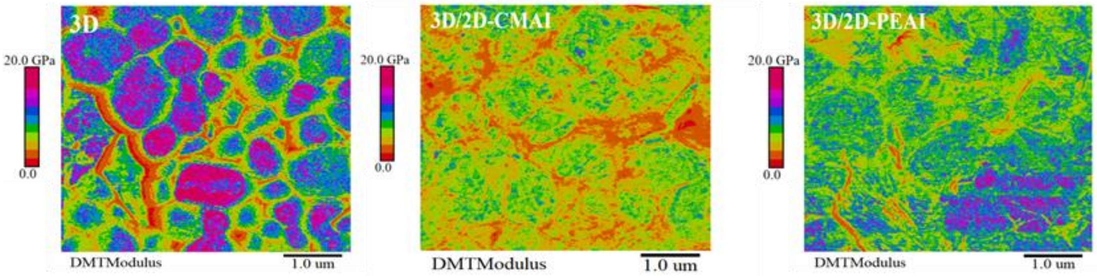


**Fig. S17** DMT modulus of pristine 3D and 3D/2D-CMAI,3D/2D-PEAI

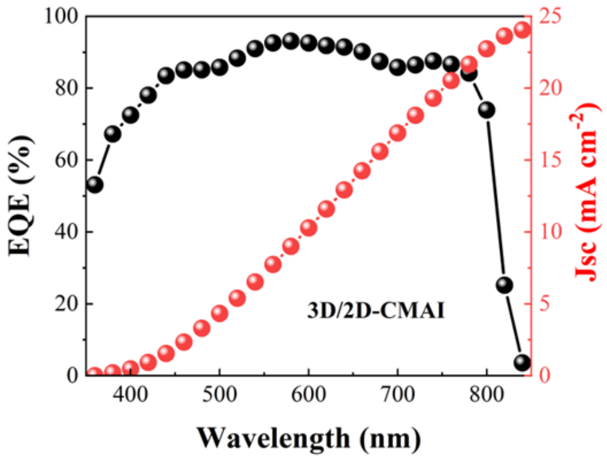


**Fig. S18** The external quantum efficiency (EQE) spectra and the integrated current density of f-PSCs

**Table S6** Summary of flexible PSC photovoltaic efficiency based on modification with CMAI

| | $V_{OC}$ (V) | $J_{SC}$ (mA cm$^{-2}$) | FF (%) | PCE (%) |
|---|---|---|---|---|
| 3D | 1.07 | 23.62 | 76.09 | 19.29 |
| 3D/2D-CMAI | 1.20 | 24.87 | 78.39 | 23.41 |

**Table S7** Summary of f-PSC photovoltaic efficiency based on 3D and 3D/2D-CMAI

| | $V_{OC}$ [a] (V) | $J_{SC}$ [a] (mA cm$^{-2}$) | FF [a] (%) | PCE [a] (%) |
|---|---|---|---|---|
| 3D | 1.06±0.005 | 22.99±0.63 | 74.56±1.53 | 18.93±0.36 |
| | (1.17) | (23.62) | (76.09) | (19.29) |
| 3D/2D-CMAI | 1.19±0.004 | 24.35±0.52 | 77.31±1.08 | 23.04±0.37 |
| | (1.20) | (24.87) | (78.39) | (23.41) |

[a] Average and standard deviation values were obtained based on over 10 cells from 2 different batches for 3D and 3D/2D-CMAI. Parameters of the best cell are reported in brackets.

**Table S8** The parameter of SCLC of Electron-only

| Electron-only | $V_{TFL}$ (V) | $n_{trap}$ (cm$^{-3}$) | μ (cm$^2$V$^{-1}$ s$^{-1}$) |
|---|---|---|---|
| 3D | 0.82 | $8.5\times10^{16}$ | $3.87\times10^{-5}$ |
| 3D/2D-PEAI | 0.58 | $5.84\times10^{16}$ | $2.07\times10^{-5}$ |
| 3D/2D-CMAI | 0.52 | $4.78\times10^{16}$ | $1.89\times10^{-5}$ |

**Table S9** The parameter of SCLC of Hole-only

| Hole-only | $V_{TFL}$ (V) | $n_{trap}$ ($cm^{-3}$) | μ ($cm^2V^{-1}s^{-1}$) |
|---|---|---|---|
| 3D | 0.62 | $6.43\times10^{16}$ | $2.43\times10^{-5}$ |
| 3D/2D-PEAI | 0.46 | $4.64\times10^{16}$ | $1.76\times10^{-5}$ |
| 3D/2D-CMAI | 0.41 | $3.77\times10^{16}$ | $1.54\times10^{-5}$ |

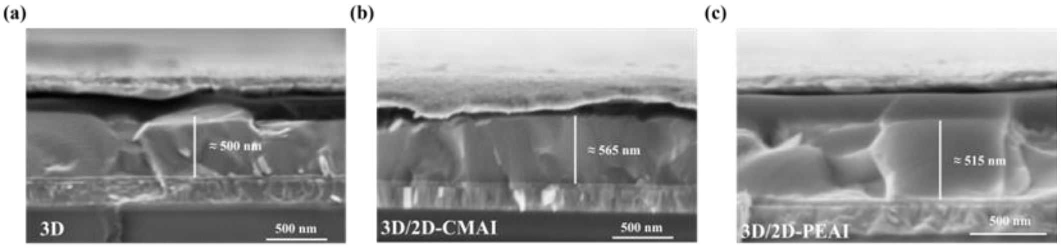


**Fig. S19** SEM cross-sectional view

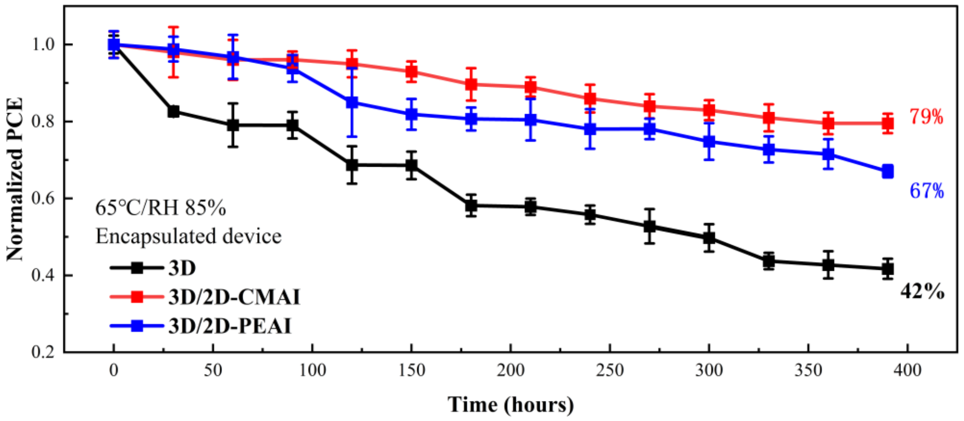


**Fig. 20** ISOS-D stability test for 3D devices and 3D/2D-CMAI,3D/2D-PEAI devices
